# Differential activities of the core planar polarity proteins during *Drosophila* wing patterning

**DOI:** 10.1016/j.ydbio.2006.09.026

**Published:** 2007-02-01

**Authors:** David Strutt, Helen Strutt

**Affiliations:** Centre for Developmental and Biomedical Genetics, Department of Biomedical Science, University of Sheffield, Western Bank, Sheffield, S10 2TN, UK

**Keywords:** Planar polarity, PCP, *Drosophila*, *Frizzled*, *Strabismus*, *Flamingo*, *Dishevelled*, *Prickle*, *Diego*

## Abstract

During planar polarity patterning of the *Drosophila* wing, a “core” group of planar polarity genes has been identified which acts downstream of global polarity cues to locally coordinate cell polarity and specify trichome production at distal cell edges. These genes encode protein products that assemble into asymmetric apicolateral complexes that straddle the proximodistal junctional region between adjacent cells. We have carried out detailed genetic analysis experiments, analysing the requirements of each complex component for planar polarity patterning. We find that the three transmembrane proteins at the core of the complex, Frizzled, Strabismus and Flamingo, are required earliest in development and are the only components needed for intercellular polarity signalling. Notably, cells that lack both Frizzled and Strabismus are unable to signal, revealing an absolute requirement for both proteins in cell–cell communication. In contrast the cytoplasmic components Dishevelled, Prickle and Diego are not needed for intercellular communication. These factors contribute to the cell–cell propagation of polarity, most likely by promotion of intracellular asymmetry. Interestingly, both local polarity propagation and trichome placement occur normally in mutant backgrounds where asymmetry of polarity protein distribution is undetectable, suggesting such asymmetry is not an absolute requirement for any of the functions of the core complex.

## Introduction

The term planar polarity was first used to describe the polarisation of structures within the plane of the insect cuticle (Nübler-Jung et al., 1987); however, the phenomenon is widespread in nature (reviewed in Klein and Mlodzik, 2005). Genetic analysis, particularly in *Drosophila*, has identified a planar polarity or PCP (planar cell polarity) pathway, dependent on the function of Frizzled (Fz) family receptors. Interestingly, not only are elements of this pathway conserved throughout the animal kingdom, but it is also required for developmental patterning processes that are distinct from planar polarity, such as polarised cell rearrangements during vertebrate gastrulation (Wallingford et al., 2002).

**Fig. 1:**
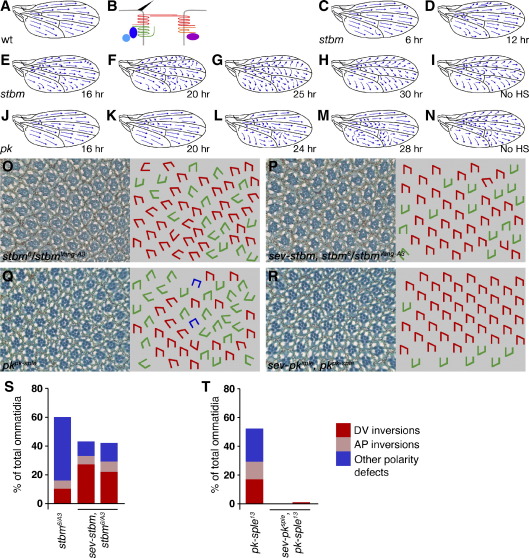
Temporal rescue of *stbm/Vang* and *pk* phenotypes in the wing and eye. All wings are shown in this and subsequent figures distal right, anterior up. Eye sections are posterior right, dorsal up. (A) Trichome polarity on the surface of a wild-type wing. (B) Cartoon showing core polarity protein distributions in the asymmetric junctional complex, Fmi/Stan in red, Fz green, Stbm/Vang orange, Dsh blue, Dgo cyan and Pk magenta. Position of trichome in black. Note that this represents an XZ section through the apicolateral junctional zone of wing cells, with distal right and apical up. (C–N) Polarity patterns in rescued wings with induction of transgene activity at indicated time. (C–I) *Act *≫ *stbm-EYFP* rescue of *stbm*^*6*^*/stbm*^*Vang-A3*^. (J–N) *Act *≫ *pk*^*pk*^ rescue of *pk*^*pk-sple-13*^. (O–R) Sections through adult eyes and cartoons. Dorsal-type ommatidia in red, ventral-type ommatidia in green, achiral ommatidia in blue. (O) *stbm*^*6*^*/stbm*^*Vang-A3*^. Ordered array is disrupted, with ommatidia pointing in random directions with randomised chirality. (P) *sev-stbm, stbm*^*6*^*/stbm*^*Vang-A3*^. Misrotation phenotype is largely rescued, but ommatidia show dorsoventral inversions due to lack of early *stbm/Vang* function. (Q) *pk*^*pk-sple-13*^. Ommatidia show random orientation and chirality. (R) *sev-pk*^*sple*^*, pk*^*pk-sple-13*^. Ommatidial polarity defect is rescued. Equator (where ommatidia change dorsoventral polarity) is at bottom of panel. (S, T) Graphs of rescue of *stbm/Vang* and *pk*. (S) Rescue of misrotation but not dorsoventral inversion phenotype in *stbm*^*6*^*/stbm*^*Vang-A3*^ with two independent *sev-stbm* transgenes. (T) Rescue of polarity defects in *pk*^*pk-sple-13*^ with two independent *sev-pk*^*sple*^ transgenes.

To date, planar polarity patterning has been best studied in the *Drosophila* wing, which provides a simple model in which each cell becomes coordinately polarised and produces a single distally pointing trichome (Fig. 1A). It is widely considered that this pattern is produced by three tiers of gene activity (Tree et al., 2002a; Klein and Mlodzik, 2005; Strutt and Strutt, 2005). At the top of the hierarchy the type II transmembrane protein Four-jointed (Fj) and the atypical cadherins Dachsous (Ds) and Fat (Ft) act (probably with other unidentified factors) to provide a long-range (or “global”) patterning cue across the axis of the tissue (Adler et al., 1998; Zeidler et al., 2000; Strutt and Strutt, 2002; Ma et al., 2003). In a manner which is not understood, but is possibly dependent on *widerborst* gene function (Hannus et al., 2002), this long-range cue is thought to be interpreted by the middle tier of genes which include *fz* and a number of other factors known as the “core” polarity genes (Shulman et al., 1998). The final tier consists of tissue-specific effectors, which modulate cellular behaviours such as polarisation of the cytoskeleton and transcription, in response to activity of components of the core.

The definition of the “core” polarity proteins is somewhat fluid, but was originally used to refer to factors that act together with Fz in all tissues examined in *Drosophila*. A notable property of Fz during planar polarity patterning is that it adopts an asymmetric subcellular localisation in polarising cells, for instance in the wing becoming localised to the junctional zone at the distal cell edge (Strutt, 2001). Five other proteins that act with Fz also adopt asymmetric localisations, either at the proximal or distal edges of wing cells, and loss of any one of these proteins prevents the distal localisation of Fz. As these proteins colocalise to junctions with Fz and are required for Fz localisation, it seems reasonable to regard them as the “core”. They consist of the multidomain cytoplasmic protein Dishevelled (Dsh) and the ankyrin repeat protein Diego (Dgo) that localise distally with Fz (Axelrod, 2001; Das et al., 2004), the fourpass transmembrane protein Strabismus (Stbm, also known as Van Gogh [Vang]) and the LIM-domain protein Prickle that localise proximally (Bastock et al., 2003; Tree et al., 2002b), and the sevenpass transmembrane cadherin Flamingo (Fmi, also known as Starry Night [Stan]) that localises both proximally and distally (Chae et al., 1999; Usui et al., 1999) (Fig. 1B). We note, that by this definition, the Gαo subunit encoded by the *brokenheart* gene may also be regarded as a component of the “core” (Katanaev et al., 2005), but this requires further investigation.

Fz is thought to perform at least three functions in planar polarity patterning. The first is to receive long-range pattering information from upstream cues, for instance provided by the activities of Fj/Ds/Ft. Experiments analysing the temporal requirements of *fz* and *ds* suggest that such coupling may occur around 6 to 24 h of pupal life (Strutt and Strutt, 2002; Matakatsu and Blair, 2004). Recent models have suggested that this information could be provided either by generation of a gradient of Fz activity across the whole axis of the wing or alternatively via generation of a gradient of Fz activity across the axis of individual cells (Lawrence et al., 2004; Amonlirdviman et al., 2005). Notably, there is currently no evidence that other components of the core are involved in this coupling.

Second, Fz is involved in a process of cell–cell communication that locally coordinates cell polarity (Adler et al., 2000; Ma et al., 2003; Lawrence et al., 2004) and also occurs after 6 h of pupal life (Strutt and Strutt, 2002). Historically, models to explain this coordination have invoked the production of a diffusible ligand for Fz (Park et al., 1994; Zheng et al., 1995; Adler et al., 1997). However, more recent models based on the observation of core polarity protein localisation to cell junctions have suggested that cell–cell signalling is contact-dependent (Tree et al., 2002b; Lawrence et al., 2004; Amonlirdviman et al., 2005; Klein and Mlodzik, 2005; Le Garrec et al., 2006). Generally, it has been assumed that all components of the core act with Fz in local coordination of polarity, but the exact roles of each protein have not been defined.

The third function of Fz is to provide a subcellular cue for trichome growth, apparently via its localisation to the distal cell edge (Wong and Adler, 1993; Strutt, 2001). In the absence of Fz, or several other core components, trichomes form in the cell centre. Provision of Fz activity after 24 h of pupal life is sufficient to permit asymmetric localisation and polarised trichome growth (Strutt and Strutt, 2002); however, distal polarity is lost, presumably due to disruption of earlier *fz* functions. As all core components asymmetrically localise together with Fz prior to trichome formation, it is tempting to conclude that all are required for trichome placement, but this has not been definitively demonstrated.

Asymmetric localisation of the core components only becomes clearly visible during pupal life by about 24 h of pupal life (but has also been observed earlier in development, see Classen et al., 2005), and hence it has been suggested that this probably follows the cell–cell communication phase (Strutt and Strutt, 2002; Lawrence et al., 2004). However, other workers have argued that asymmetric complex formation may occur progressively over a longer period of pupal life, and be intrinsically required for cell–cell communication and local coordination of polarity (Tree et al., 2002b; Amonlirdviman et al., 2005). In this context, it is important to consider that the spatial relationships observed during asymmetric complex formation (Fz, Dsh, Dgo and Fmi/Stan colocalising at distal cell edges; Stbm/Vang, Pk and Fmi/Stan at proximal edges) may not necessarily reflect earlier functional relationships. Notably, associations have also been reported between Dsh and Pk (Tree et al., 2002b; Jenny et al., 2005), Dsh and Stbm/Vang (Bastock et al., 2003; Jenny et al., 2005), Dgo and Pk (Das et al., 2004) and Dgo and Stbm/Vang (Das et al., 2004).

In this manuscript, we address three key issues: First, which components of the core act together with Fz during the different planar polarity patterning processes? Second, are the spatial relationships seen during the later phase of asymmetric localisation also relevant during the phase of cell–cell communication and local coordination of polarity? Third, is asymmetric core protein localisation absolutely required for planar polarity patterning?

## Materials and methods

### Fly strains and genetics

Alleles and transgenes used are described in FlyBase, except where noted. Temporal rescue of polarity phenotypes in the wing and eye was carried out and analysed as described (Strutt and Strutt, 2002). *Actin* ≫ *fz-EYFP* and *Actin* ≫ *stbm-EYFP* have been described (Strutt, 2001; Strutt et al., 2002), *Actin* ≫ *dsh-ECFP*, *Actin* ≫ *fmi-FLAG*, *Actin* ≫ *pk*^*pk*^, *sev-stbm* and *sev-pk*^*sple*^ were constructed as previously (Strutt and Strutt, 2002). Note that the *pk* locus produces two protein isoforms, of which the Pk variant is sufficient for wing patterning and the Sple variant is sufficient for eye patterning (Gubb et al., 1999). For double mutant clones, rescue of *fz* activity on the X and 2R was provided by *Arm-fz-EGFP* transgenes (Strutt, 2001) and rescue of *stbm/Vang* activity on the X was provided by an *Actin-stbm-EYFP* transgene. *fz;stbm* twinclones were generated by inducing clones of *FRT42 stbm*^*6*^ *Arm-fz-EGFP* in a *fz* background, resulting in cells homozygous for *stbm*^*6*^ *Arm-fz-EGFP* juxtaposed to twinspot cells lacking the transgene. Clones in the wing were generally induced using *Ubx-FLP*, kindly provided by Jürgen Knoblich.

Exact genotypes used are as follows:

**Figure 1**

Temporal rescue of *stbm/Vang* in wing: *w hsFLP1; stbm*^*6*^*/stbm*^*Vang-A3*^*; Act-FRT-polyA-FRT-stbm-EYFP/+*
Temporal rescue of *pk*^*pk-sple*^ in wing: *w hsFLP1; pk*^*pk-sple-13*^*/pk*^*pk-sple-13*^*; Act-FRT-polyA-FRT-pk/+*
stbm/Vang phenotype in eye: *w; FRT42 stbm*^*6*^*/FRT42 P[w*^*+*^*] stbm*^*Vang-A3*^
Rescue of *stbm/Vang* phenotype in eye by *sev-stbm*: *w; FRT42 stbm*^*6*^*/FRT42 P[w*^*+*^*]stbm*^*Vang-A3*^*; sevE-sevP-stbm*^*7.1*^*/+*
*pk*^*pk-sple-13*^ phenotype in eye: *FRT42 pk*^*pk-sple-13*^ *cn sp/FRT42 pk*^*pk-sple-13*^ *cn*
Rescue of *pk*^*pk-sple*^ phenotype in eye by *sev-pk*^*sple*^: *w; FRT42 pk*^*pk-sple-13*^ *cn/FRT42 pk*^*pk-sple-13*^ *cn; sevE-sevP-sple*^*2.2*^*/+*
Genotypes shown in graph in (S): *w; stbm*^*6*^*/FRT42 P[w*^*+*^*] stbm*^*Vang-A3*^ *w; FRT42 stbm*^*6*^*/FRT42 P[w*^*+*^*] stbm*^*Vang-A3*^*; sevE-sevP-stbm*^*2.2*^*/+*, *w; FRT42 stbm*^*6*^*/FRT42 P[w*^*+*^*] stbm*^*Vang-A3*^*; sevE-sevP-stbm*^*7.1*^*/+*
Genotypes shown in graph in (T): *FRT42 pk*^*pk-sple-13*^ *cn/FRT42 pk*^*pk-sple-13*^ *cn* *w; FRT42 pk*^*pk-sple-13*^ *cn/FRT42 pk*^*pk-sple-13*^ *cn; sevE-sevP-pk*^*sple[2.2]*^*/+* *w; FRT42 pk*^*pk-sple-13*^ *cn/FRT42 pk*^*pk-sple-13*^ *cn; sevE-sevP-pk*^*sple[14.2]*^*/+*

**Figure 2**

*fz* clones using rescuing transgene on 2R: *w; FRT42D/FRT42D Arm-fz-EGFP, Arm-lacZ; fz*^*15*^*/fz*^*21*^*, Ubx-FLP*
*stbm/Vang* clones: *y w Ubx-FLP/+; FRT42D stbm*^*6*^*/FRT42D Arm-lacZ*
*stbm/Vang*; *fz* double clones: *w; FRT42D stbm*^*6*^*/FRT42D Arm-fz-EGFP; fz*^*21*^*/fz*^*21*^*, Ubx-FLP*
*stbm/Vang* and *fz* twin clones: *w; FRT42D stbm*^*6*^*, Arm-fz-EGFP/FRT42D Arm-lacZ; fz*^*21*^*/fz*^*21*^*, Ubx-FLP*
*fmi/stan* clones: *y w Ubx-FLP/+; FRT42D fmi*^*E59*^*/FRT42D Arm-lacZ*
*fmi/stan*; *fz* double clones: *w; FRT42D fmi*^*E59*^*/FRT42D Arm-fz-EGFP, Arm-lacZ; fz*^*21*^*/fz*^*21*^*, Ubx-FLP*
*stbm/Vang fmi/stan* double clones: *y w Ubx-FLP/+; FRT42D stbm*^*6*^ *fmi*^*E59*^*/FRT42D Arm-lacZ*

**Figure 3**

*dsh*^*3*^ clones: *y w dsh*^*3*^ *FRT18A/w Arm-lacZ FRT18A; FLP38/+dsh; fz* double clones:
*y w dsh*^*3*^ *f*^*36a*^ *FRT19A/w Arm-fz-EGFP FRT19A; fz*^*21*^*/fz*^*21*^*, Ubx-FLP*
*dsh; stbm/Vang* double clones: *y w dsh*^*3*^ *f*^*36a*^ *FRT19A/w Act-FRT-polyA-FRT-stbm-EYFP FRT19A; stbm*^*6*^*/stbm*^*6*^*, Ubx-FLP*
*pk*^*pk-sple*^ clones: *y w Ubx-FLP/+; FRT42D pk*^*pk-sple-13*^*/FRT42D Arm-lacZ*
*pk*^*pk-sple*^*; fz* double clones: *w; FRT42D pk*^*pk-sple-13*^*/FRT42D Arm-fz-EGFP; fz*^*21*^*/fz*^*21*^*, Ubx-FLP*
*pk*^*pk-sple*^*stbm/Vang* double clones: *y w Ubx-FLP/+; FRT42D pk*^*pk-sple-13*^ *stbm*^*6*^*/FRT42D Arm-lacZ*

**Figure 4**

*stbm/Vang dgo* double clones: *y w Ubx-FLP/+; FRT42D stbm*^*6*^ *dgo*^*380*^*/FRT42D Arm-lacZ*
*pk*^*pk-sple*^*dgo* double clones: *y w Ubx-FLP/+; FRT42D pk*^*pk-sple-13*^ *dgo*^*380*^*/FRT42D Arm-lacZ*
*pk*^*pk-sple*^*dgo; fz* triple clones: *w; FRT42D pk*^*pk-sple-13*^ *dgo*^*380*^*/FRT42D Arm-fz-EGFP; fz*^*21*^*/fz*^*21*^*, Ubx-FLP*

**Figure 5**

*stbm/Vang* overexpression in *fz* background: *y w hsFLP1/+; Act-FRT-y*^*+*^*-FRT-GAL4, UAS-lacZ/+; fz*^*15*^*, UAS-stbm/Df(3L)fz*^*D21*^
*fz-EGFP* overexpression in *stbm/Vang* background: *w hsFLP1/+; Act-FRT-y*^*+*^*-FRT-GAL4, stbm*^*6*^*/stbm*^*6*^*, UAS-fz-EGFP*
*fz-EGFP* overexpression in *pk*^*pk-sple*^ background: *w hsFLP1/+; Act-FRT-y*^*+*^*-FRT-GAL4, pk*^*pk-sple-13*^*/pk*^*pk-sple-13*^*, UAS-fz-EGFP*
*fz-EGFP* overexpression in *pk*^*pk-sple*^ *dgo*^*380*^ background: *w hsFLP1/+; FRT42D pk*^*pk-sple-13*^ *dgo*^*380*^*/FRT42D pk*^*pk-sple-13*^ *dgo*^*380*^*; Act-FRT-CD2-FRT-GAL4, UAS-fz-EGFP/+*
*fz-EGFP* overexpression in *dsh*^*1*^: *w dsh*^*1*^*/Y; FLP38/Act-FRT-y*^*+*^*-FRT-GAL4, UAS-lacZ; UAS-fz/+*
*fz-EGFP* overexpression under *ptc-GAL4* control in wings containing *dsh*^*3*^ clones: *y w dsh*^*3*^ *f*^*36a*^ *FRT19A/y w w*^*+*^*FRT19A; ptc-GAL4/+; UAS-fz-EGFP, Ubx-FLP/+*

**Figure 6**

*Act-fz-EYFP* expression in *pk*^*pk-sple*^ background: *w hsFLP1/+; pk*^*pk-sple-13*^*, Act-FRT-polyA-FRT-fz-EYFP/pk*^*pk-sple-13*^
*Act-fz-EYFP* expression in *dsh*^*1*^ background: *w dsh*^*1*^*/Y; Act-FRT-polyA-FRT-fz-EYFP/FLP38*
*dgo* clones: *y w Ubx-FLP/+; FRT42D dgo*^*380*^*/FRT42D Arm-lacZ*
*pk*^*pk-sple*^ clones: *y w Ubx-FLP/+; FRT42D pk*^*pk-sple-13*^*/FRT42D Arm-lacZ*
*pk*^*pk-sple*^*dgo* double clones: *y w Ubx-FLP/+; FRT42D pk*^*pk-sple-13*^ *dgo*^*380*^*/FRT42D Arm-lacZ*
*dsh*^*3*^ clones: *y w dsh*^*3*^ *FRT18A/w Arm-lacZ FRT18A; FLP38/+*

**Supplementary Fig. 1:**
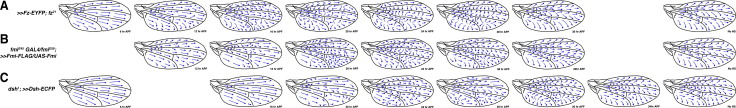
Temporal rescue of *fz*, *fmi/stan* and *dsh*^*1*^ phenotypes in the wing. Diagrams of adult wings are shown distal to the right and anterior up. Trichome polarity patterns are shown for rescue of the mutant phenotype by induction of ubiquitous transgene expression by heat-shock at the indicated time. (A) *Act* ≫ *fz-EYFP* rescue of the null allele *fz*^*21*^. Apart from minor defects in the proximal wing, induction at 6 h APF rescues the *fz* phenotype to a wild-type polarity pattern. Rescues at progressively later timepoints up to 28 h result in phenotypes of increasing severity, which differ both qualitatively and quantitatively from the unrescued loss-of-function phenotype (“No HS”). We have previously attributed this stronger than loss-of-function phenotype to a loss of only the non-autonomous functions of *fz* which act to coordinate polarity locally between cells, whilst retaining the later autonomous function which is required for trichome placement at the cell edge (Strutt and Strutt, 2002). Induction at 30 h, APF gives the same polarity pattern as no induction and corresponds to the normal loss-of-function phenotype produced by loss of the autonomous function, indicating that *fz* is absolutely required for autonomous function and asymmetric trichome placement prior to this time. (B) *Act* ≫ *fmi-FLAG* rescue of *fmi*^*E45*^*/fmi*^*E59*^. The embryonic lethality of loss of *fmi/stan* activity is rescued by *fmi/stan* expression in the embryonic nervous system from *UAS-Fmi* as previously described (Usui et al., 1999). Heat-shocking of prepupae of this genotype at 0 h APF and 6 h APF resulted in lethality for unknown reasons. However, induction of transgene expression at 12 h APF provided partial rescue of the *fmi/stan* trichome polarity defect. Progressively later induction up to 28 h APF again resulted in phenotypes of increasing severity as seen for *fz* rescue, which differ from the unrescued loss-of-function phenotype. We again infer that as for *fz*, this may be due to loss of local coordination of polarity, but retention of a later autonomous function required for asymmetric trichome placement. Induction at 30 h, APF results in a trichome pattern resembling the unrescued loss-of-function phenotype. (C) *Act* ≫ *dsh-ECFP* rescue of *dsh*^*1*^. Induction of transgene expression at 6 h APF results in complete rescue of the *dsh* trichome polarity phenotype, and almost complete rescue is seen for induction at 16 h APF. Induction at 20 h APF results in moderate defects in several regions of the wing, which are nevertheless much weaker than the defects in *fz*, *stbm/Vang* or *fmi/stan* wings rescued at this timepoint. Rescue at 24 to 30 h produces strong defects that differ from the unrescued loss-of-function defect. We speculate that this could be caused by sudden induction of Dsh protein production perturbing the polarity of existing asymmetric complexes that already contain the transmembrane proteins Fz, Stbm/Vang and Fmi/Stan. Induction at 34 h APF (after trichome formation) has no effect on trichome polarity, as would be expected.

**Supplementary Figure 1**

Temporal rescue of *fz*: *y w hsFLP1; Act-FRT-polyA-FRT-fz-EYFP/+; fz*^*21*^
Temporal rescue of *fmi/stan*: *y w hsFLP1; fmi*^*E45*^*, GAL4-1407/fmi*^*E59*^*; Act-FRT-polyA-FRT-fmi-FLAG/UAS-fmi*
*GAL4-1407* and *UAS-fmi* provide rescue of *fmi* activity in the embryonic nervous system (Usui et al., 1999)
Temporal rescue of *dsh*: *w dsh*^*1*^*/Y; FLP38/+; Act>FRT-poly-FRT-dsh-ECFP/+*

**Supplementary Fig. 2:**
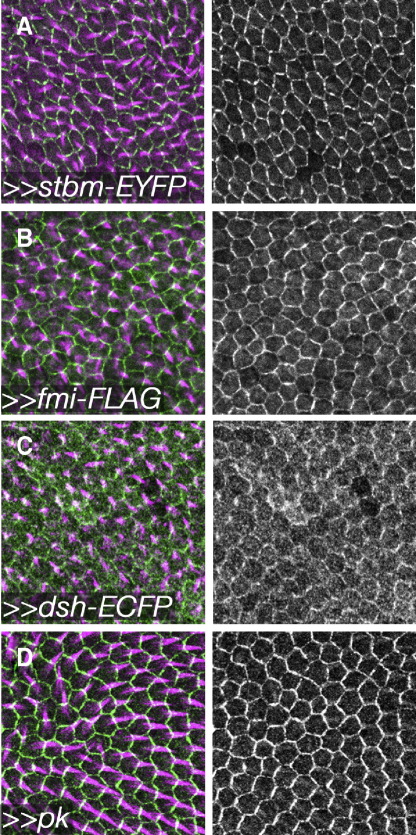
Localisation of induced proteins and asymmetric trichome placement. Immunostaining of wings with transgene expression induced at 24 h, dissected at 32 h, showing actin (magenta) and polarity protein distribution (green in left panel, white in right panel) stained for GFP (A, C), Fmi/Stan (B) and Pk (D), respectively. (A) Induction of *stbm-EYFP* expression in a *stbm*^*6*^*/stbm*^*Vang-A3*^ background. Almost all cells express detectable Stbm-EYFP, which localises asymmetrically to cell edges, with polarised trichomes forming at the site of localisation. (B) Induction of *fmi-FLAG* expression in a *fmi*^*E45*^*/fmi*^*E59*^ background. Fmi-FLAG is seen localised at cell edges, corresponding to the site of polarised trichome initiation. (C) Induction of *dsh-ECFP* expression in a *dsh*^*1*^ background. Almost all cells express detectable levels of Dsh-ECFP. Although this fusion protein appears to localise relatively poorly, it nevertheless exhibits asymmetric localisation at sites of polarised trichome formation. (D) Induction of *pk*^*pk*^ expression in a *pk*^*pk-sple-13*^ background. Asymmetrically localised Pk protein can be seen in all cells, corresponding to the sites of polarised trichome production.

**Supplementary Figure 2**

*w hsFLP1; stbm*^*6*^*/stbm*^*Vang-A3*^*; Act-FRT-polyA-FRT-stbm-EYFP/+*
y *w hsFLP1; fmi*^*E45*^*, GAL4-1407/fmi*^*E59*^*; Act-FRT-polyA-FRT-fmi-FLAG/UAS-fmi*
*w dsh*^*1*^*/Y; FLP38/+; Act>FRT-poly-FRT-dsh-ECFP/+*
*w hsFLP1; pk*^*pk-sple-13*^*/pk*^*pk-sple-13*^*; Act-FRT-polyA-FRT-pk/+*

**Supplementary Fig. 3:**
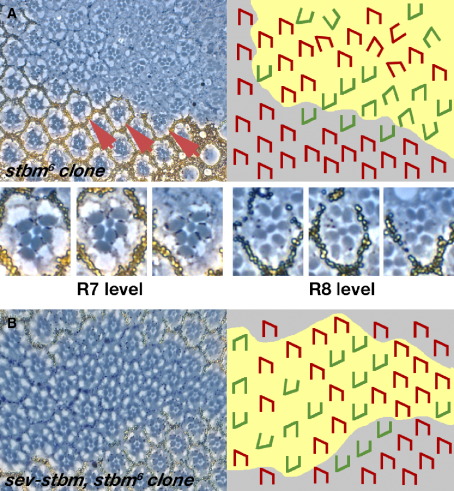
Non-autonomous phenotypes of *stbm/Vang* clones in the adult eye. Sections through adult eyes (dorsal up, posterior right) containing clones of cells homozygous for *stbm*^*6*^, marked by lack of pigment (brown). Cartoons on right show mutant tissue in yellow, dorsal-type ommatidia in red, ventral-type ommatidia in green. (A) *stbm*^*6*^ clone. Ommatidial polarity is randomised within the clone, with both rotation and dorsoventral chirality defects evident. On the equatorial edge of the clone (bottom edge in picture), inversions of ommatidial dorsoventral polarity are seen extending into wild-type tissue (arrows). Hence *stbm/Vang* exhibits both autonomous (within the clone) and non-autonomous (outside the clone) ommatidial polarity defects in the eye. Insets in lower part of panel show high magnification images of the three inverted ommatidia indicated by the arrows, taken from sections at both R7 and R8 photoreceptor level. Note dark pigment granules in all seven photoreceptors in the R7 level section, and in the stalk of the R8 photoreceptors (entering between R1 and R2) in the R8 level section, indicating that all 8 photoreceptors retain *stbm/Vang* activity. In total we examined 18 *stbm*^*6*^ clones and 16 *stbm*^*15*^ clones for evidence of non-autonomy of the *stbm/Vang* ommatidial polarity phenotype. Out of this set of 34 clones, we found 6 clones (3 each for *stbm*^*6*^ and *stbm*^*15*^) which had at least one ommatidium on the equatorial edge which was phenotypically mutant (i.e. showed inverted dorsoventral polarity) but genotypically wild-type for all 8 photoreceptors (9 ommatidia in total). Hence we conclude that *stbm/Vang* does show non-autonomy of the ommatidial polarity phenotype, albeit in only a relatively small subset of clones. This result stands in contrast to the findings of a previous analysis of the genotypes of at least 170 misoriented ommatidia on the edges of *stbm/Vang* clones, which concluded that the *stbm/Vang* polarity phenotype was autonomous (Wolff and Rubin, 1998; T. Wolff personal communication). We suggest two possible reasons for this discrepancy. Firstly, our data set might be more likely to reveal a low rate of ommatidial non-autonomy than the one previously analysed, by virtue of the way that we selected clones for analysis. As we were predisposed to the hypothesis that *stbm/Vang* clones might show equatorial non-autonomy of the polarity phenotype (by analogy to the proximal non-autonomy seen in the wing Taylor et al., 1998), we deliberately selected for analysis large clones with equatorial boundaries that were separated from the equator of the eye. Hence, even if *stbm/Vang* non-autonomy is only rarely seen, we will have greatly increased our chances of observing the phenomenon, as compared to the previously used data set in which misoriented ommatidia on all edges of clones in positions throughout the eye were examined (Wolff and Rubin, 1998). It should be noted that a low rate of ommatidial non-autonomy for the *stbm/Vang* phenotype is not unexpected, given that in the wing the range of non-autonomy around *stbm/Vang* clones only extends about 5–10 cells and varies depending upon clone position in the wing (Taylor et al., 1998; Adler et al., 2000). To see non-autonomy at the level of a whole ommatidium, all of the precursor cells for the 8 photoreceptors would have to lie within 5–10 cells of the equatorial edge of a *stbm/Vang* clone, a situation which would probably only occur rather infrequently. A second, less attractive, reason for the discrepancy may be that some of the stocks used to generate the clones have acquired either enhancers or suppressors of the *stbm/Vang* non-autonomous phenotype. We think this unlikely, as the chromosomes used in our study and the previous study were all generated in the same screen (Wolff and Rubin, 1998), and we see the non-autonomy with two different chromosomes—but we cannot rule out this possibility. (B) *stbm*^*6*^ clone in an eye also expressing *stbm* under control of the *sevenless* promoter, which is specifically expressed in differentiating photoreceptors. Expression of *stbm* under *sevenless* control is able to substantially rescue the ommatidial rotation defects within the clone, but not the dorsoventral polarity defects, suggesting that *stbm/Vang* has a function prior to photoreceptor differentiation which is required specifically to determine dorsoventral polarity. Note again that ommatidia with dorsoventral polarity defects are seen in wild-type tissue on the equatorial edge of the clone, indicating a non-autonomous role for *stbm/Vang* function in the eye.

**Supplementary Figure 3**

*stbm/Vang* clones: *w hsFLP1; FRT42 stbm*^*6*^*/FRT42 P[w*^*+*^*]*
*stbm/Vang* clones rescue by *sev-stbm*: *w hsFLP1; FRT42 stbm*^*6*^*/FRT42 P[w*^*+*^*]; sevE-sevP-stbm*^*7.1*^*/+*

Note that *fz*^*21*^, *stbm*^*6*^, *dsh*^*3*^, *fmi*^*E59*^, *pk*^*pk-sple-13*^ and *dgo*^*380*^ have been molecularly characterised and are thought to be null alleles on the basis of being unable to give rise to functional proteins (Jones et al., 1996; Wolff and Rubin, 1998; Wehrli and Tomlinson, 1998; Usui et al., 1999; Gubb et al., 1999; Feiguin et al., 2001). *fmi*^*E45*^ contains a missense mutation that generates an amorphic mutation in the wing by genetic criteria (Usui et al., 1999). *fz*^*15*^ contains a nonsense mutation that gives rise to a truncated protein that has been characterised as amorphic in the wing (Jones et al., 1996). *stbm*^*Vang-A3*^ has not been molecularly characterised, but has been defined by genetic criteria to be amorphic in the wing (Taylor et al., 1998). *dsh*^*1*^ contains a missense mutation in the DEP domain which has been reported to be a strong mutation for planar polarity functions of the gene (Perrimon and Mahowald, 1987; Axelrod et al., 1998; Boutros et al., 1998).

### Histology

Pupal wings were processed for immunofluorescence and imaged as previously (Strutt, 2001). Primary antibodies used for experiments or confirmation of genotypes were mouse monoclonal anti-βgal (Promega), rabbit anti-βgal (Cappel), rabbit anti-GFP (Abcam), mouse monoclonal anti-Fmi#74 (DSHB, Usui et al., 1999), rabbit anti-Pk (Tree et al., 2002b), rabbit anti-Stbm (Rawls and Wolff, 2003), rat anti-Dsh (Shimada et al., 2001) and rabbit anti-Dgo (Feiguin et al., 2001). Actin was visualised using Texas-Red-conjugated phalloidin (Molecular Probes). Adult wings were mounted in GMM and eye sections were prepared as described (Tomlinson and Ready, 1987).

## Results

### Differing temporal requirements of the core polarity proteins during wing development

We previously analysed the temporal requirements of *fz* for planar polarity patterning in the wing, by rescuing the phenotype of *fz* mutant flies using an inducible *fz*-expressing transgene (Strutt and Strutt, 2002). Expression of the transgene is activated at different times during pupal development, by administration of a heat-shock, allowing determination of the latest timepoint that gene expression is sufficient to permit normal patterning. These studies found no requirement for *fz* function prior to 6 h after prepupa formation (APF). Progressively later heat-shocks up to 24 h APF produced stronger phenotypes that were qualitatively and quantitatively different from the reported *fz* loss-of-function phenotype. We classified this stronger phenotype as *ds*-like, as Fz protein was still localising at cell edges and specifying the site of trichome formation, but due to a loss of non-autonomous coordination of polarity Fz localisation was seen in a swirling pattern rather than uniformly at distal cell edges (Strutt and Strutt, 2002). Heat-shocks after 28 h APF resulted in the reported *fz* loss-of-function phenotype, consistent with this being produced by loss of the later autonomous function that places trichomes at the cell edge.

As the core polarity gene *stbm/Vang* shows similar phenotypes to *fz*, exhibiting both strong domineering non-autonomous effects on trichome polarity and being required for trichome placement at the cell edge (Taylor et al., 1998), we considered it a good candidate for sharing common functions with *fz*. Using the same methodology, we analysed its timecourse of requirement in wing patterning (Figs. 1C–I). In common with *fz*, *stbm/Vang* is not required prior to 6 h APF, but then shows progressively stronger phenotypes when induced between 12 and 24 h APF, with induction at 30 h APF mimicking the normal loss-of-function phenotype (seen when no heat-shock is administered, Fig. 1I). For comparison, we repeated our analysis of the timecourse of *fz*-requirement (Supplementary Fig. 1A), but this time using the molecularly characterised *fz*^*21*^ null allele (Jones et al., 1996). This gave the same timecourse as observed for *stbm/Vang*, although generally with slightly stronger phenotypes being observed.

Next we analysed the temporal requirement of the core polarity gene *pk*, which produces a protein that colocalises with Stbm/Vang at the proximal cell edge and which has been implicated in cell–cell coordination of planar polarity (Tree et al., 2002b). Interestingly, induction of *pk* expression as late as 20 h APF resulted in only negligible polarity defects in the adult wing, with induction at 24 h and 28 h still providing partial rescue of *pk* function (Figs. 1J–N).

These results indicate that whereas *stbm/Vang* shares an early requirement with *fz* in the wing, *pk* has only a relatively late function. We further extended these results by investigating the requirements of the other two core components *fmi/stan* and *dsh* (Supplementary Figs. 1B, C). To circumvent the embryonic lethality of *dsh* null alleles, we analysed rescue of the strong planar polarity phenotype of the viable *dsh*^*1*^ allele (Perrimon and Mahowald, 1987) (the core component dgo was not examined, as the adult wing phenotype is too subtle for this approach to be feasible, Feiguin et al., 2001).

Induction of *fmi/stan* expression between 12 and 24 h APF resulted in progressively stronger phenotypes that differ from the loss-of-function phenotype (Supplementary Fig. 1B), as observed for *fz* and *stbm/Vang*. Conversely, induction of *dsh* at 16 to 20 h APF resulted in relatively minor defects, although later induction revealed a strong requirement for *dsh* function after 20 h APF (Supplementary Fig. 1C). Hence, *fmi/stan* appears to share early requirements with *fz* and *stbm/Vang*, whereas *dsh* exhibits later temporal requirements. However, we cannot rule out the possibility that the *dsh*^*1*^ allele exhibits residual activity in planar polarity, which might contribute to the apparently later requirement.

For all genotypes, early transgene induction can rescue, indicating that the transgenes provide appropriate levels of expression throughout the wing. Consistent with this, almost all cells express detectable protein after transgene induction (Supplementary Fig. 2). Furthermore, without induction, we observe the expected loss-of-function phenotype seen in the absence of the transgene, indicating that our results are unlikely to be due to “leaky” expression from the transgenes. We tested whether the differences might be due to transmembrane proteins taking longer to be synthesised and targeted to the appropriate subcellular sites; however, we found that after induction both Fz-EYFP and Dsh-ECFP show the appearance of junctional staining within 2–3 h (data not shown).

### Differing temporal requirements of the core polarity proteins during eye development

We also find a common early requirement for *fz* and *stbm/Vang* in the eye. We previously distinguished between early and late activities of *fz* in the eye, by expressing *fz* under control of the *sevenless* promoter which is not active until the time of photoreceptor differentiation (Strutt and Strutt, 2002). Providing *fz* activity only at the time of photoreceptor differentiation resulted in defects in the dorsoventral polarity of ommatidia, indicating that *fz* activity is specifically required prior to photoreceptor differentation for correct specification of dorsoventral polarity. However, lack of *fz* activity after photoreceptor differentiation results in randomisation of all aspects of ommatidial polarity including both dorsoventral and anteroposterior polarity and rotation. Hence, *fz* shows two phases of requirement during eye development, an early phase needed just for dorsoventral patterning and a later phase required for dorsoventral and anteroposterior polarity and rotation of ommatidia.

In contrast, we showed that ommatidial polarity and rotation defects within null mutant *dsh*^*3*^ tissue can be rescued completely by expression of *sev-dsh*, indicating that *dsh* does not share the early dorsoventral patterning function with *fz*, but nevertheless is required for the later phase of activity.

We have now extended this work to *stbm/Vang* and *pk*. Rescuing the phenotype of *stbm/Vang* in the eye by expression of *sev-stbm* reduces general polarity and rotation defects but reveals an underlying randomisation of dorsoventral polarity (Figs. 1O, P, S) indicating that *stbm/Vang* shares with *fz* an early dorsoventral patterning function.

Conversely, the *pk* phenotype is almost completely rescued by a *sev-pk*^*sple*^ transgene (Figs. 1Q, R, T) which expresses the Sple isoform of the Pk protein which is specifically required for eye patterning (Gubb et al., 1999). Thus *pk*, like *dsh*, does not exhibit an early patterning function in eye development.

Interestingly, while investigating the functions of *stbm/Vang* in the eye, we found that *stbm/Vang* clones also show equatorial non-autonomy of the polarity phenotype. In a number of cases we observed dorsoventral polarity inversions in ommatidia on the equatorial sides of clones, in which all 8 photoreceptors of the ommatidium retain *stbm/Vang* activity (Supplementary Fig. 3). This is consistent with the observed non-autonomy of clones in the wing (Taylor et al., 1998). However, a previous analysis of over 169 misoriented ommatidia on the edges of clones found no significant evidence of non-autonomy of the polarity phenotype of *stbm/Vang* (Wolff and Rubin, 1998). Re-examination of this original data set in the light of our results again failed to find evidence of non-autonomy (T. Wolff, personal communication). The reasons for this discrepancy are currently unclear (but see legend to Supplementary Fig. 3 for further discussion of this issue).

### Mutual dependence of *fz*, *stbm/Vang* and *fmi/stan* for intercellular communication

**Fig. 2:**
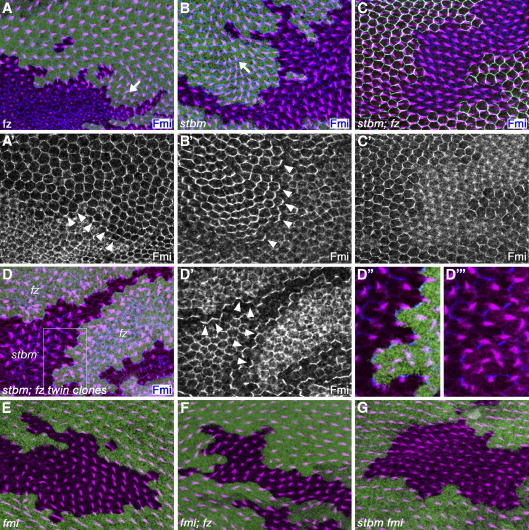
*fz*, *stbm/Vang* and *fmi/stan* activities are essentially required for intercellular polarity signalling. 32 h pupal wings stained for actin (magenta), clonal marker (*lacZ* or *fz-EGFP*, green) and Fmi/Stan (blue in A–D, D′′,D‴; white in A′–D′). Arrows indicate direction of abnormal trichome polarity around clones. Arrowheads indicate localised Fmi/Stan at sites of polarised trichome formation on clone boundaries. (A, A′) *fz*^*15*^*/fz*^*21*^ mutant clone, generated using *Arm-fz-EGFP* rescuing transgene on 2R. Arrow indicates orientation of abnormally polarised trichomes pointing towards mutant tissue. (B, B′) *stbm*^*6*^ clone. Arrow indicates orientation of abnormally polarised trichomes pointing away from mutant tissue. (C, C′) *stbm*^*6*^*; fz*^*21*^ double clone. Note trichomes in non-mutant tissue are normally polarised. (D, D′) *stbm*^*6*^ and *fz*^*21*^ twin clones. Higher magnification view of boxed region in panel D shown in panels D′′ and D‴, note production of polarised trichomes pointing into *fz* tissue at sites of localised Fmi/Stan. (E) *fmi*^*E59*^ clone. Note that trichomes in non-mutant tissue are normally polarised. (F) *fmi*^*E59*^*; fz*^*21*^ double clone. Note that trichomes in non-mutant tissue are normally polarised. (G) *stbm*^*6*^*fmi*^*E59*^ double clone. Note trichomes in non-mutant tissue are normally polarised.

The common early function of *fz*, *stbm/Vang* and *fmi/stan* is likely to be either receiving long-range patterning cues and/or local coordination of polarity. Little is understood about how the long-range signal might be received, rendering this activity difficult to study. However, the effects of *fz* and *stbm/Vang* on local coordination of polarity can be assayed, as groups of cells lacking the activity of either gene cause neighbouring cells to mispolarise: *fz* clones cause neighbouring cells to point their trichomes towards the clone (arrow Fig. 2A, Gubb and García-Bellido, 1982; Vinson and Adler, 1987), whereas trichomes point away from *stbm/Vang* clones (arrow Fig. 2B, Taylor et al., 1998).

As during later pupal life Fz and Stbm/Vang are seen localised to adjacent cell boundaries (Strutt, 2001; Bastock et al., 2003), it has been proposed that polarity coordination requires signals to pass between Fz and Stbm/Vang expressing cells. Some evidence for this has been presented in the abdomen (Lawrence et al., 2004), and recent models for planar polarity coordination in the wing are based on this hypothesis (Amonlirdviman et al., 2005; Klein and Mlodzik, 2005; Le Garrec et al., 2006). However, there is no rigorous experimental evidence for signals passing between Fz and Stbm/Vang expressing cells in the wing. Furthermore, if such signalling does occur, it is not known whether signals might pass monodirectionally from Fz to Stbm/Vang, monodirectionally from Stbm/Vang to Fz or bidirectionally.

To address this issue, we generated clones of cells simultaneously mutant for both *fz* and *stbm/Vang*. We reasoned that if signals pass strictly monodirectionally from Stbm/Vang to Fz, then wild-type cells outside of a clone would receive the same aberrant polarity cue from a *stbm/Vang; fz* double mutant clone as from a *stbm/Vang* single mutant clone. Thus, *stbm/Vang*; *fz* double mutant clones should show the same polarity phenotype as *stbm/Vang* single mutant clones.

Conversely, if signals pass strictly from Fz to Stbm/Vang, cells outside should polarise as if neighbouring a *fz* clone and not a *stbm/Vang* clone. In this case, cells require Fz to send polarity cues and cells mutant for both *fz* and *stbm/Vang* provide the same aberrant polarity cue as cells mutant for only *fz*.

However, if there is a bidirectional interaction, such that cells expressing Fz need to contact cells expressing Stbm/Vang and vice versa, then the result is harder to predict. In this case, signal receiving cells would require both Fz and Stbm/Vang and similarly signal sending cells would require both Fz and Stbm/Vang. Hence, one possibility is that clones of cells doubly mutant for *fz* and *stbm/Vang* would send or receive no polarity signals, and thus might have no effect on the polarity of their neighbours. A precedent for this prediction has been provided by work in the abdomen, where experimental results suggest that cells that lack Fmi/Stan are unable to send or receive polarity cues, and in this case the neighbours to exhibit normal polarity (Lawrence et al., 2004). However, it is also possible that a failure to send or receive cues might result in neighbouring cells adopting a randomised polarity.

Control clones lacking only *fz* activity (marked by lack of *lacZ* expression) show trichomes pointing towards the clone (arrow Fig. 2A); whereas *stbm/Vang* clones (marked by lack of *lacZ*) show trichomes pointing away (arrow Fig. 2B). We then generated double mutant *stbm/Vang*; *fz* clones using null alleles of both *fz* and *stbm/Vang* and an *Arm-fz-EGFP* transgene (which rescues fz activity, see Strutt, 2001) located on the same chromosome arm as *stbm/Vang*. This resulted in genetically mosaic wings containing clones of cells of the genotype *stbm*^*6*^*/stbm*^*6*^*; fz*^*21*^*/fz*^*21*^ juxtaposed to twinspot tissue of the genotype *Arm-fz-EGFP/Arm-fz-EGFP; fz*^*21*^*/fz*^*21*^ or heterozygous tissue of the genotype *Arm-fz-EGFP/stbm*^*6*^*; fz*^*21*^*/fz*^*21*^ (see Materials and methods). Such clones of *stbm/Vang; fz* cells (marked by lack of Fz-EGFP, green) show negligible effects on the polarity of trichomes in neighbouring cells (Fig. 2C, trichomes visualised by labelling for Actin, magenta). This result fits the hypothesis that bidirectional interactions occur between Fz and Stbm/Vang expressing cells, and that lack of communication with cells within a clone leads to neighbouring cells adopting a wild-type polarity.

Interestingly, within the double mutant clones, trichome polarity is also relatively unperturbed (Fig. 2C). This is in contrast to single mutant *fz* and *stbm/Vang* clones, where trichomes of a sufficient age adopt polarities consistent with those shown by trichomes outside the clone (e.g. Fig. 2B). We do not fully understand this phenomenon; however, it is well-established that trichomes within *fz* and *stbm/Vang* tissue largely emerge in the cell centre without obvious polarity (Wong and Adler, 1993; Taylor et al., 1998). We surmise that such “apolar” trichomes subsequently align themselves with the strongly polarised trichomes in the wild-type tissue surrounding the clone- possibly as a result of cytoskeletal interactions between adjacent cells.

We also examined the distribution of the core polarity protein Fmi/Stan on the boundaries of clones of cells singly or doubly mutant for *fz* and *stbm/Vang*. It has previously been shown that Fmi/Stan strongly localises to the boundaries between *fz*^+^ and *fz*^−^ tissue and *stbm/Vang*^+^ and *stbm/Vang*^−^ tissue (arrowheads Figs. 2A′, B′; Usui et al., 1999; Bastock et al., 2003). Although not formally proven, it is widely thought that such localised protein localisation might mediate cell–cell communication (Amonlirdviman et al., 2005; Klein and Mlodzik, 2005; Le Garrec et al., 2006). Consistent with this view, there is no strong localisation of Fmi/Stan on the boundaries of *stbm/Vang; fz* double clones (Fig. 2C′).

So far our results suggest that Fz in one cell and Stbm/Vang in the adjacent cell is necessary for cell–cell communication and polarisation of trichomes. We next investigated whether Fz and Stbm/Vang in adjacent cells were sufficient for this process. To do this, we examined the effect of juxtaposing cells that lack *fz* activity to cells that lack *stbm/Vang* activity. This was achieved by generating clones homozygous for the genotype *FRT42D stbm*^*6*^*, Arm-Fz-EGFP; fz*^*21*^ juxtaposed to twinspots of the genotype *FRT42D Arm-lacZ; fz*^*21*^ (see Materials and methods), such that cells lacking *stbm/Vang* activity also lacked *lacZ* expression, whereas cells lacking both *fz* activity and the rescuing *Arm-fz-EGFP* transgene exhibited high levels of *lacZ* expression.

On the boundaries where *stbm/Vang* tissue is juxtaposed to *fz* tissue, we observe strong Fmi/Stan localisation (Fig. 2D, arrowheads in Fig. 2D′) resembling that seen on the edges of *fz* or *stbm/Vang* clones (Figs. 2A′,B′). Notably, at the edges of *fz* and *stbm/Vang* clones, localised Fmi/Stan is associated with production of polarised trichomes (Figs. 2A, B), apparently as a result of assembly of a polarised asymmetric core polarity protein complex with Fz on one side of the cell–cell boundary and Stbm/Vang on the other side (Fig. 1B). We also observe polarised trichomes produced at the site of Fmi/Stan localisation on boundaries between *stbm/Vang* and *fz* tissue, which point towards the *fz* tissue (Figs. 2Dʺ,D‴). Taken together, the localisation of Fmi/Stan and the production of polarised trichomes suggest that a functional core polarity protein complex assembles on the boundaries between *stbm/Vang* and *fz* tissue and that this complex is sufficient to specify polarised trichome formation.

We note that within the *stbm/Vang* and *fz* mutant tissue, there is no assembly of asymmetric complexes and trichome placement is unpredictable (Figs. 2D, D′), as expected from previous work (Wong and Adler, 1993; Taylor et al., 1998).

We next analysed the role of Fmi/Stan in cell–cell communication of polarity cues in the wing. Unlike *fz* or *stbm/Vang* clones, clones of cells lacking *fmi/stan* activity do not strongly affect the polarity of neighbouring cells (Fig. 2E, Chae et al., 1999; Usui et al., 1999). This could be interpreted to suggest that *fmi/stan* is not required for cell–cell communication and coordination of cell polarity. However, the Fmi/Stan protein is thought to act as a homophilic cell adhesion molecule (Usui et al., 1999) and so alternatively Fmi/Stan may be required in both sending and receiving cells for coordination of cell polarity and loss of *fmi/stan* blocks cell–cell communication. Support for this second view comes from experiments in the abdomen, where cells overexpressing Fz or Stbm/Vang are unable to repolarise their neighbours if they also lack *fmi/stan* activity (Lawrence et al., 2004).

We generated clones of cells double mutant for either *fz* and *fmi/stan* or for *stbm/Vang* and *fmi/stan*. Both showed a phenotype typical of single mutant *fmi/stan* clones (Figs. 2F, G), indicating that Fmi/Stan is required in both Fz and Stbm/Vang expressing cells for cell–cell communication, and thus by extension is required on both sides of the cell–cell boundary.

Thus, for the *fz*-dependent process of cell–cell communication that is thought to locally coordinate cell polarity, we have demonstrated that Fz and Stbm/Vang are required in opposite cells and that Fmi/Stan is required in both cells. This spatial arrangement is as seen in the asymmetric complex that assembles at the site of trichome initiation (Fig. 1B), and supports models in which a subset of this complex is also involved in intercellular communication.

### dsh and pk are not required for intercellular communication

In the late asymmetric complex, Dsh associates with Fz at distal cell edges whereas Pk localises with Stbm/Vang at proximal cell edges. Hence, it is possible that the association of Dsh with Fz is essential for signalling to Stbm/Vang in the adjacent cell, and similarly that Pk association with Stbm/Vang is required for signalling to Fz in the adjacent cell. However, interactions have also been reported between Dsh and Pk and between Dsh and Stbm/Vang (Tree et al., 2002b; Bastock et al., 2003; Jenny et al., 2005), and during an early phase of cell–cell communication these interactions might also be important.

**Fig. 3:**
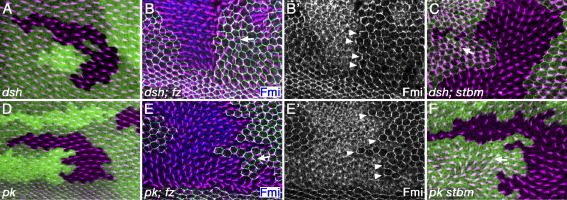
*dsh* and *pk* are not required for intercellular signalling. 32 h pupal wings stained for actin (magenta), clonal marker (*lacZ*, *stbm-EYFP* or *fz-EGFP*, green) and Fmi/Stan (blue in B, E; white in B′, E′). Arrows indicate direction of abnormal trichome polarity around clones. Arrowheads indicate localised Fmi/Stan at sites of polarised trichome formation on clone boundaries. (A) *dsh*^*3*^ clone. Note that trichomes in non-mutant tissue are normally polarised. (B, B′) *dsh*^*3*^*; fz*^*21*^ double clone. Note that abnormally polarised trichomes pointing towards the mutant tissue (arrow) and accumulation of Fmi/Stan on clone boundary (arrowheads). (C) *dsh*^*3*^*; stbm*^*6*^ double clone. Arrow indicates orientation of abnormally polarised trichomes pointing away from mutant tissue. (D) *pk*^*pk-sple-13*^ clone. Note that trichomes in non-mutant tissue are normally polarised. (E, E′) *pk*^*pk-sple-13*^*; fz*^*21*^ double clone. Note abnormally polarised trichomes pointing towards the mutant tissue (arrow) and accumulation of Fmi/Stan on clone boundary (arrowheads). (F) *pk*^*pk-sple-13*^*stbm*^*6*^ double clone. Arrow indicates orientation of abnormally polarised trichomes pointing away from mutant tissue.

Clones of cell mutant for *dsh* alone do not strongly affect the polarity of neighbouring cells (Fig. 3A, Theisen et al., 1994), in this respect resembling *fmi/stan* clones. Thus, like *fmi/stan*, it is possible that *dsh* might be required on both sides of cell–cell boundaries for communication to occur, consistent with its known physical associations with both Fz and Stbm/Vang. However, we found that both *dsh*; *fz* and *dsh*; *stbm/Vang* double mutant clones showed non-autonomous effects on the polarity of neighbouring cells, typical of either single mutant *fz* or *stbm/Vang* clones respectively (arrows Figs. 3B, C), with normal accumulation of Fmi/Stan at clone boundaries (arrowheads Fig. 3B′). These results demonstrate that *dsh* activity is not required for Fz-Stbm/Vang-dependent intercellular communication to occur, in either Fz-expressing or Stbm/Vang-expressing cells.

We carried out similar experiments using a null allele of *pk*^*pk-sple*^. Single mutant clones of *pk* also do not significantly affect the polarity of neighbouring cells (Fig. 3D, Gubb et al., 1999). However, *pk-sple*; *fz* and *pk-sple stbm/Vang* double mutant clones still show the typical non-autonomous effects of *fz* or *stbm/Vang* clones respectively (arrows Figs. 3E, F) and accumulation of Fmi/Stan at clone boundaries (arrowheads Fig. 3E′). We conclude that *pk* activity is also not essentially required for Fz-Stbm/Vang-dependent intercellular communication.

The range of non-autonomous alterations in cell polarity around *fz* and *stbm/Vang* clones is generally up to 10 cell diameters; however, it varies with clone size, shape and position (Vinson and Adler, 1987; Taylor et al., 1998; Adler et al., 2000). We observed similar ranges of non-autonomy for the double mutant clones generated with null *dsh* and *pk* alleles, suggesting that the strength of intercellular signalling remained in the normal range.

### Analysis of dgo function

**Fig. 4:**
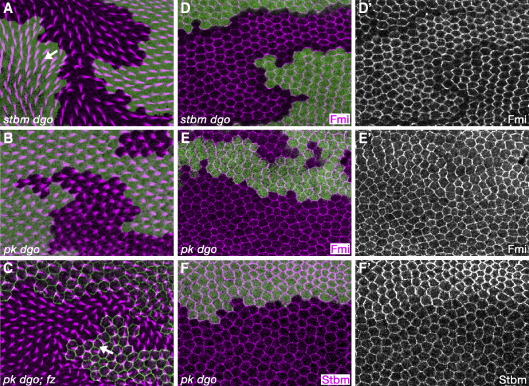
*dgo* is not redundant with *pk* or *stbm/Vang* for intercellular signalling or apicolateral polarity protein localisation. (A–C) 32 h pupal wings stained for actin (magenta). (D–F) 28 h pupal wings stained for Fmi/Stan or Stbm/Vang (magenta or white in separation). Clonal markers in green (*lacZ* or *fz-EGFP*). Arrows indicate direction of abnormal trichome polarity around clones. (A, D) *stbm*^*6*^*dgo*^*380*^ double clones. Arrow indicates orientation of abnormally polarised trichomes pointing away from mutant tissue. (B, E, F) *pk*^*pk-sple-13*^*dgo*^*380*^ double clones. (C) *pk*^*pk-sple-13*^*dgo*^*380*^*; fz*^*21*^ triple clone. Arrow indicates orientation of abnormally polarised trichomes pointing towards mutant tissue.

It has been recently reported that during planar polarity patterning of the *Drosophila* eye disc, the core polarity gene *dgo* acts redundantly with *stbm/Vang* and *pk* (Das et al., 2004). If such a situation also pertained in the wing, we reasoned that this might mask specific roles of either *pk* or *dgo* in intercellular signalling. However, we find that *stbm/Vang dgo* clones still exhibit the proximal non-autonomy typical of *stbm/Vang* single clones (arrow Fig. 4A) and *pk dgo* clones behave like *pk* single clones, showing no non-autonomous effect on trichome polarity (Fig. 4B). In an attempt to test the hypothesis of redundant functions of core polarity proteins as rigorously as possible, we generated clones of cells triply mutant for *pk*, *dgo* and *fz*. These also behaved like single mutant *fz* clones, showing typical non-autonomous effects on trichome polarity (arrow Fig. 4C).

In the eye, *dgo* has been particularly implicated in cooperating with *pk* and *stbm/Vang* to maintain the junctional localisation of Fmi/Stan (Das et al., 2004). Interestingly, in the pupal wing Fmi/Stan remains junctional in *stbm/Vang dgo* clones (Fig. 4D), although proximodistal asymmetric localisation is lost as previously reported for *stbm/Vang* clones (Bastock et al., 2003). The same is true of Fmi/Stan localisation in *pk dgo* clones (Fig. 4E). We also find that contrary to the reported situation in the eye, Stbm/Vang apicolateral localisation is maintained in *pk dgo* clones (Fig. 4F).

### Polarity defects propagate through tissue mutant for *pk*, *pk dgo* and *dsh* to different degrees

Mosaic experiments so far described have analysed the ability of clones of cells lacking the function of one or more core polarity genes to influence the polarity of neighbouring cells via intercellular signalling. However, for polarity defects to propagate away from such clones, intracellular signalling is required across the axes of individual cells, in addition to intercellular signalling between cells. The nature of intracellular signalling is poorly understood. One recent model proposes that it depends upon detection of levels of intracellular *fz* activity by transmembrane receptors on different sides of the cell (Lawrence et al., 2004), whereas others suggest that it relies on asymmetric assembly of protein complexes on one cell edge in response to the presence of an asymmetric complex at the opposite cell edge (Amonlirdviman et al., 2005; Klein and Mlodzik, 2005; Le Garrec et al., 2006).

Although *dsh*, *pk* and *dgo* do not have essential functions in intercellular signalling, they might nevertheless be required for intracellular communication. However, in this context, it is interesting to note that polarity defects can still propagate around *fz* clones in abdomens wholly mutant for a null allele of *pk* (Lawrence et al., 2004) and in wings mutant for the *pk*^*1*^ mutation which mutates one of the *pk* isoforms (Adler et al., 2000). Taken at face value, these data suggest that *pk* may not be essentially required for either intercellular or intracellular signalling.

To investigate this further, we generated clones of cells with altered *fz* or *stbm/Vang* activity in wings wholly mutant for the function of other core polarity genes. As it simplified the generation of the appropriate fly strains, in these experiments we generated clones of cells with increased Fz activity (which cause neighbouring trichomes to point away from the clone, see e.g. Strutt, 2001) or Stbm/Vang activity (which cause trichomes to point towards the clone, Amonlirdviman et al., 2005).

**Fig. 5:**
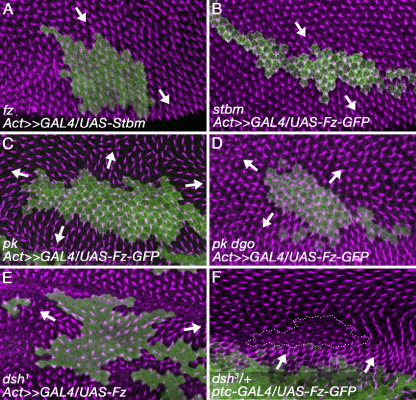
Propagation of polarity defects in polarity gene mutant backgrounds. 32 h pupal wings stained for actin (magenta) and clonal marker (*lacZ* or *fz-EGFP*, green). Arrows indicate direction of trichome polarity around clones. (A) Stbm/Vang overexpressing clone in *fz*^*15*^*/Df(3L)fz*^*D21*^. Note trichome polarity is not altered by clone (arrows). (B) Fz-EGFP overexpressing clone in *stbm*^*6*^. Note trichome polarity is not altered by clone (arrows). (C) Fz-EGFP overexpressing clone in *pk*^*pk-sple-13*^. Trichomes point away from clone (arrows). (D) Fz-EGFP overexpressing clone in *pk*^*pk-sple-13*^*dgo*^*380*^. Trichomes point away from clone (arrows). (E) Fz overexpressing clone in *dsh*^*1*^. Trichomes point away from clone (arrows). (F) Clone of *dsh*^*3*^ cells (outlined by white dots) in wing overexpressing Fz-EGFP (green) at anteroposterior compartment boundary. Trichomes point away from region of Fz-EGFP expression (arrows).

For control experiments, we analysed wings entirely mutant for *fz* or *stbm/Vang* activity. These factors are required both for intercellular communication and trichome placement at the cell edge, so overexpression clones in these backgrounds are not expected to alter the polarity of neighbouring cells (e.g. Taylor et al., 1998). Consistent with this, clones of cells that overexpress Stbm/Vang cannot alter trichome polarity in *fz* wings (Fig. 5A), and clones of cells overexpressing Fz cannot alter trichome polarity in *stbm/Vang* wings (Fig. 5B).

However, if Fz is overexpressed in clones of cells in wings wholly mutant for a null allele of *pk*, we observed a strong effect on trichome polarity in neighbouring cells (Fig. 5C), which extends 8–9 cells (average 8.6, *n* = 11) from the clone boundary, similar to the effect seen in wild-type wings and in agreement with previous observations (Adler et al., 2000; Lawrence et al., 2004). Interestingly, in wings double mutant for *pk* and *dgo*, clones of cells overexpressing Fz also affect the polarity of neighbouring cells (Fig. 5D), but the effect only extends for 5–6 cells (average 5.6, n = 8). Hence, although patterning is essentially normal in *dgo* wings, and polarity defects propagate a normal distance though *pk* tissue, when both factors are absent the propagation of polarity defects is substantially reduced, revealing an unexpected redundancy between these factors for this process.

We also investigated the effect of overexpressing Fz in clones of cells in wings mutant for *dsh*. To circumvent the lethality of *dsh* null mutations, we used the *dsh*^*1*^ point mutation which affects only planar polarity functions. We again observed an effect of the clones on the polarity of neighbouring cells (Fig. 5E); however, in this case, polarity defects only propagated at most 3–4 cells from the clone (average 3.7, *n* = 7). Finally, we tested propagation through cells mutant for a null allele of *dsh*. Non-autonomous polarity defects were induced by overexpressing Fz-GFP in the *patched* expression domain at the anteroposterior compartment boundary (Adler et al., 1997), in wings containing *dsh*^*3*^ clones (Fig. 5F). Within the clones, we observed trichomes emerging largely in the cell centre as previously reported (Wong and Adler, 1993), and were unable to detect propagation of polarity defects.

### Asymmetry of polarity protein activity exists in the absence of asymmetry in distribution

**Fig. 6:**
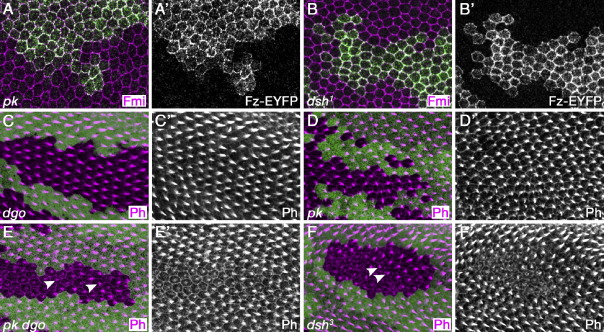
(A, B) 28 h pupal wings stained for Fmi/Stan (magenta) or Fz-EYFP (green or white in separation). (C–F) 32 h pupal wings stained for actin (magenta). Clonal marker in green (*lacZ*). Arrowheads indicate trichomes emerging in cell centre. (A) Clone of cells expressing Fz-EYFP under the *Actin5C* promoter in *pk*^*pk-sple-13*^. (B) Clone of cells expressing Fz-EYFP under the *Actin5C* promoter in *dsh*^*1*^. (C) *dgo*^*380*^ clone. Trichomes form at distal cell edges in cells inside the clone at the same time as in cells outside the clone. (D) *pk*^*pk-sple-13*^ clone. Trichomes form at distal cell edges in cells inside the clone at the same time as in cells outside the clone. (E) *pk*^*pk-sple-13*^*dgo*^*380*^ double clone. Trichome formation is delayed inside the clone and is often in cell centre (arrowheads). (F) *dsh*^*3*^ clone. Trichome formation is delayed inside the clone and is often in cell centre (arrowheads).

Recent models have suggested that asymmetric localisation might be essential for polarity propagation (Amonlirdviman et al., 2005; Klein and Mlodzik, 2005; Le Garrec et al., 2006). This is at variance with observations of propagation of polarity defects in *pk* or *dsh*^*1*^ backgrounds (Figs. 5C, E and Adler et al., 2000; Lawrence et al., 2004), in which asymmetry is not observed (Figs. 6A, B and e.g. Axelrod, 2001; Strutt, 2001; Usui et al., 1999). However, it is possible that weak asymmetry exists in these backgrounds, which is difficult to detect. We investigated this more closely by generating clones of cells expressing Fz-EYFP. In general, a subtle increase or decrease in Fz-EYFP at the edge of one cell might be masked by Fz-EYFP localisation on the adjacent boundary of a neighbouring cell (as it is not possible to distinguish between localisation at adjacent cell boundaries by visible light microscopy). However, by looking at Fz-EYFP distribution on the edges of clones, it should be easier to discern subtle asymmetry of proximodistal localisation (see Strutt, 2001). Hence we examined the distribution of Fz-EYFP expressed in clones in null *pk* and *dsh*^*1*^ backgrounds, but still were unable to observe any evidence of asymmetric localisation (Figs. 6A′,B′).

Notwithstanding our failure to find asymmetry of distribution of polarity proteins in *pk* and *dsh*^*1*^ wings, we nevertheless suppose that there must be asymmetry of activity in order for propagation of polarity and asymmetric trichome placement to occur. Interestingly, in clones of cells lacking *dgo* or *pk* activity, trichome formation occurs both at the same time as in adjacent non-mutant tissue and at the distal cell edge (Figs. 6C, D). Hence in these backgrounds there is clearly an asymmetric signal for trichome formation. In contrast, in clones of cells doubly mutant for *dgo* and *pk*, trichome formation in often seen to be delayed and when it does occur is often seen in the cell centre (Fig. 6E), indicative of loss of the asymmetric trichome placement cue. The phenotype in *pk dgo* clones is similar to that seen in tissue mutant for *dsh*, *fz* or *stbm/Vang* (Fig. 6F, Wong and Adler, 1993; Taylor et al., 1998). Hence, consistent with the differing effects of *dsh*, *pk* and *dgo* on propagation of polarity defects, we see a similar progression of effects on the asymmetric placement of trichomes at the cell edge, suggesting that these two processes are linked.

## Discussion

As described in the Introduction, it is possible to define a core group of polarity proteins, but this does not imply that all components of the core make equal contributions to planar polarity patterning. In this work, we have attempted to systematically analyse the contributions of the core proteins to the processes of coupling to the global cue, local coordination of polarity and asymmetric trichome placement.

Our key findings are as follows:

(i) The transmembrane proteins Fz, Stbm/Vang and Fmi/Stan have a common early function during planar polarity patterning in the wing and eye, with the cytoplasmic factors Dsh and Pk playing only later roles.
(ii) The transmembrane core of Fz, Fmi/Stan and Stbm/Vang is absolutely required for intercellular communication. We demonstrate that the asymmetric relationship of these proteins seen at the time of trichome placement, with Fmi/Stan in both communicating cells and Fz in one cell juxtaposed to Stbm/Vang in the adjacent cell, is also necessary and sufficient for such intercellular communication. In addition we provide evidence that information passes both from Fz to Stbm/Vang expressing cells *and* vice versa.
(iii) Intercellular communication does not require Dsh or Pk in either Fz or Stbm/Vang expressing cells and Dgo also does not play redundant roles with Pk in intercellular signalling.
(iv) Pk and Dgo act redundantly in propagation of polarity from cell to cell, most likely by promoting intracellular communication. Dsh plays a prominent role in such propagation, greater than that of both Pk and Dgo. We speculate that the intracellular communication required for such polarity propagation is dependent on establishing intracellular asymmetries of protein activity.
(v) Although even subtle asymmetry of Fz localisation is not apparent in *pk* tissue, not only does polarity propagate between cells, but trichome placement also occurs at the normal time and place. Hence, asymmetry of polarity protein activity exists in the absence of detectable asymmetry of localisation.
(vi) Neither Pk nor Dgo are directly required for determining the site of trichome placement.

### Coupling to the global polarity cue

As noted in the Introduction, one of the putative functions of the core polarity proteins is to couple to long-range polarity patterning cues. It has been suggested that these cues are provided by *fj/ds/ft* (Ma et al., 2003; Yang et al., 2002), but little is understood regarding the molecular mechanism of any such coupling. We suppose that *fz*, *stbm/Vang* and *fmi/stan* are involved as they show the earliest requirement. Comparison with the temporal requirements of *ds* (Matakatsu and Blair, 2004), argues against a role for *pk*, and probably *dsh* (with the caveat that we were unable to analyse a null allele). Interestingly, it has been argued that during abdomen patterning, *pk* may play a particular role in “rectifying” the global signal in different compartments (Lawrence et al., 2004). In the wing, such a function is not necessary, possibly explaining why we do not find a corresponding early role for *pk*.

### Local coordination of polarity

A better understood and major function of Fz and the core polarity proteins is the local coordination of cell polarity. All recent models for this coordination have proposed a role for cell–cell contact mediated signalling, as opposed to schemes requiring the secretion of a diffusible ligand. A key feature of such models is that they require both intercellular communication to pass polarity cues between adjacent cells and intracellular communication to pass information across the axes of individual cells. Experimental support for cell–cell contact mediated signalling has been provided by experiments in the abdomen, showing that the atypical cadherin Fmi/Stan is required in both signal sending and receiving cells, suggesting that signals pass between Fmi/Stan homodimers (Lawrence et al., 2004). Theoretical evidence has also been provided by a number of mathematical models that have confirmed the feasibility of locally coordinating polarity via assembly of asymmetric junctional complexes containing Fz in one cell and Stbm/Vang in the adjacent cell (Amonlirdviman et al., 2005; Le Garrec et al., 2006).

Our results rigorously demonstrate that, in the wing, intercellular signalling events that locally coordinate polarity require Fmi/Stan in both communicating cells and Fz in one cell and Stbm/Vang in the other. This supports models in which the asymmetric junctional distributions that are observed by immunofluorescence are required for intercellular signalling (Amonlirdviman et al., 2005; Klein and Mlodzik, 2005; Le Garrec et al., 2006). However, it should be noted that although signalling may require the assembly of complexes with Fz in one cell adjacent to Stbm/Vang in the next, this does not necessarily imply that detectable asymmetric subcellular distribution of proteins within cells is necessary. Indeed the persistence of signalling in *pk* and *dsh*^*1*^ backgrounds where subcellullar asymmetry of the core polarity proteins is not observed argues against this being essential. In addition, our data raise the possibility of bidirectional cell–cell communication via Fz-Stbm/Vang, and are inconsistent with a monodirectional signal as proposed to occur in the abdomen (Lawrence et al., 2004).

It is also evident from our results that intercellular signalling does not require association of Dsh, Pk or Dgo with Fz or Stbm/Vang. Indeed, *fz* or *stbm/Vang* clones that also lack any of these factors are not obviously impaired in their ability to alter the polarity of neighbouring cells.

What then are the roles of Dsh, Pk and Dgo? We show that propagation of polarity defects away from a clone is reduced in *dsh* and *pk dgo* tissue, indicating that they are required for local relay of polarity cues. Hence, a likely role would be in the intracellular signalling required to pass polarity cues across the axes of cells. Previous experiments in which polarity was seen to propagate normally in a *pk* background (Adler et al., 2000; Lawrence et al., 2004) argued against such a function for *pk*, which is only revealed when *dgo* function is also absent.

One proposed mechanism for intracellular signalling is that each cell acquires a particular level of Fz activity, which is communicated by intercellular signalling to all surrounding cells (Lawrence et al., 2004). In this case, roles for Dsh, Pk and Dgo in modulating intracellular levels of Fz activity could be envisaged. However, the majority of models suggest roles for Dsh and Pk in intracellular feedback loops that amplify differences in the asymmetric localisation of the core polarity protein complexes within cells (Amonlirdviman et al., 2005; Klein and Mlodzik, 2005; Le Garrec et al., 2006). For instance, it has been proposed that association of Dsh with Fz might be antagonised by high local concentration of Stbm/Vang-Pk (Amonlirdviman et al., 2005), or alternatively that Fz-Dsh antagonise Stbm/Vang-Pk interactions (Le Garrec et al., 2006). In either case, if asymmetric complexes containing Fz, Fmi/Stan and Stbm/Vang were somehow stabilised by addition of Dsh, Pk and/or Dgo to the complex, then such antagonistic interactions would provide feedback that would amplify asymmetries of protein localisation across the axes of individual cells. Notably, our experimental results suggest that if such feedback is occurring, then the relative importance of the cytoplasmic factors in stabilising complex formation follows the hierarchy Dsh > Pk > Dgo. Such a scheme would also explain the redundant functions of *pk* and *dgo*, even though these factors act at opposite cell edges, as Pk on one side of a cell–cell boundary could bind to and stabilise a complex that was also being stabilised by Dgo binding on the opposite side of the cell–cell boundary. Simultaneous loss of both Pk and Dgo would have a greater destabilising effect than loss of either factor alone.

However, models that depend on amplification of differences in asymmetric subcellular protein distribution have to be reconciled with the failure to observe protein asymmetries in *pk* or *dsh*^*1*^ tissue, through which polarity can still propagate. Possibly in these backgrounds there are subtle asymmetries which cannot be observed—notably at least one recent model predicts such subtle asymmetry in *pk* clones (Amonlirdviman et al., 2005). But another explanation is that receptor proteins such as Fz may be uniformly distributed, but nevertheless exhibit differential signalling activity across the axes of cells.

Despite the apparently non-essential role of protein asymmetry either in polarity propagation over short distances or in trichome placement, it nevertheless seems likely that it is an active mechanism in ensuring robust coordination of polarity and correct trichome placement over the whole wing (Ma et al., 2003; Amonlirdviman et al., 2005), as otherwise the failure of long-range coordination of polarity in *pk* wings cannot be explained. Interestingly, it has recently been reported that asymmetry is present from as early as the third instar stage of development, but is subsequently lost during junctional remodelling in pupal stages (Classen et al., 2005), suggesting that such asymmetry could be playing a role from much earlier in development than previously suspected.

### Specification of the site of polarised trichome production

The precise mechanism by which asymmetric trichomes are generated remains unknown, although there appears to be a role for asymmetric subcellular activities of polarity effector proteins such as Inturned (Adler et al., 2004). As asymmetric trichomes can be generated in the absence of Pk or Dgo, it seems unlikely that either of these proteins interacts directly with the trichome placement machinery; however, all of the other core polarity proteins are candidates for such a role.

### Concluding remarks

In conclusion, we note that there has recently been great interest in attempting to mathematically model the processes underlying propagation of planar polarity between cells (e.g. Lawrence et al., 2004; Amonlirdviman et al., 2005; Klein and Mlodzik, 2005; Le Garrec et al., 2006). Although the presented models have been very successful at reproducing known phenomena, they have nevertheless been based on limited experimental data. This work both provides support for some of the assumptions of such models, for instance by directly testing the central role of core transmembrane proteins such as Fz, Fmi/Stan and Stbm/Vang in intercellullar signalling, but also provides challenges, for instance by demonstrating the propagation of polarity in the absence of visible protein asymmetry.
